# Coping and back problems: analysis of multiple data sources on an entire cross-sectional cohort of Swedish military recruits

**DOI:** 10.1186/1471-2474-7-39

**Published:** 2006-05-03

**Authors:** Charlotte Leboeuf-Yde, Kristian Larsen, Ingvar Ahlstrand, Ernest Volinn

**Affiliations:** 1 Research Professor, The Back Research Center, Part of Clinical Locomotion Science, University of Southern Denmark, Lindevej 5, DK-5750 Ringe, Denmark; 2 Researcher, The Musculoskeletal Research Unit, Orthopaedic Department, Holstebro Hospital, Lægaardsvej 12 Holstebro DK-Holstebro, Denmark; 3 Statistician Pliktverket Karolinen Se-65180 Karlstad Sweden; 4 Research Associate Professor Pain, Research Center University of Utah School of Medicine, Department of Anesthesiology 615 Arapeen Drive, Suite 200 Salt Lake City, Utah 84108, USA

## Abstract

**Background:**

As the literature now stands, a bewildering number and variety of biological, psychological and social factors are, apparently, implicated in back problems. However, if and how these have a direct influence on back problems is not clear. Obesity, for example, has in many studies been shown to be associated with back problems but there is no evidence for a causal link. This could be explained by a dearth of suitably designed studies but also because obesity may be but a proxy for some other, truly explanatory variable. Coping has been linked with, particularly, persistent back problems as well as with health in general. The question is, whether coping could be the explanatory link between, for example, these two variables. A cross-sectional study was undertaken using data from the Swedish Army, consisting of the entire cohort of males (N = 48,502) summoned in 1998 to serve in the military. The purpose of the study was to investigate the relation between five independent variables and two dependent variables ("outcome variables"). The independent variables were two anthropomorphic variables (height and body mass index), two psychological variables (intellectual capacity and coping in relation to stress), and one social variable (type of education). The two outcome variables were back problems and ill health. In particular, we wanted to determine whether controlling for coping would affect the associations between the other four independent variables and the two outcome variables.

**Methods:**

Data for the analysis come from a battery of standardized examinations, including medical examinations, a test of intellectual capacity, and a test of coping in relation to stress. Each of these examinations was conducted independently of the others. Unadjusted and adjusted odds ratios were calculated for the outcome variables of back problems and ill health.

**Results:**

The associations between height, body mass index, intellectual capacity, type of education and the two outcome variables (back problems and ill health) were weak to moderate. Additionally, there were strong associations between coping and the two outcome variables and when controlling for coping the previously noted associations diminished or disappeared, whereas none of the other variables had a large effect on the association between coping and the two outcome variables.

**Conclusion:**

Coping emerged as strongly associated with both back problem and ill health and coping had a leveling effect on the associations between the other independent variables and the two outcome variables. This study is noteworthy particularly because the association with coping is so robust. It is a retrospective, cross-sectional study, however, and, as such it raises questions of causality; which – if any – came first, inability to cope or back pain? The results of this study call attention to the need for a prospective study, in which coping is clearly defined. Such a study has been undertaken and will be presented separately.

*Index terms: *back pain, coping, education, height, BMI, intellectual capacity, bio-psycho-social model, epidemiology, cohort, cross-sectional study

## Background

For more than a generation, researchers have called upon the "biopsychosocial model" to explain disease in general [1]. In therapeutic circles, most back patients are probably, at least initially, considered from a pathoanatomical aspect, and in particular unsuitable ergonomic circumstances and excessive workload are considered relevant obstacles to recovery. Increasingly, however, psychosocial factors have come to outweigh biological factors in explanations of back pain, in particular for persistent back pain [2]. Even so, the relative importance of particular bio-psycho-social factors requires further studies. For example, smoking [3] and obesity [4] have been shown in many studies to be positively associated with back problems but it is not known whether this link is causal or an expression of some other underlying biological or psychological variable, which is linked with both smoking/obesity and back problems.

One factor that could be suspected of having such a dual link, coping, has attracted attention. People vary in their ability to cope with stress and their coping strategies differ as well [5]. Research in this area is extensive but has not resulted in a useful classification system, and the clinical validity of the concept is as yet unclear [6]. Coping is variously defined in the literature, and the literature on it is voluminous. Nevertheless, the classical definition of "coping", one at the core of most others, is "purposeful efforts to manage or modify the negative impact of stress" [7]. Comprehensive reviews have shown that coping in its various guises is related to the onset of chronic pain and outcomes of treatment for it [18]. Specifically regarding back problems, however, the literature is both sparse and conflicting [9-11]. We were interested in examining whether coping was associated with back problems and whether coping could explain some associations between various potential risk factors and back problems often reported in the literature.

Social class is another factor that is often implicated in ill health. Intellectual capacity and type of education can be considered proxy measures of social class, because both these would lead to greater or lesser societal opportunities and occupational exposures. In order to establish a possible link between these factors and back problems, it would be necessary to study a young population that has not yet been subjected to occupational exposures. Again, it would be interesting to see if coping plays an independent role in relation to back problems when studied in relation to these two basic determinants of social class.

Another issue to be taken into account in the explanation of back problems is co-morbidity. Studies of adults [12] as well as of children have shown that those with back problems are more likely also to suffer from other disorders [13]. This repeated finding raises another critical question: Are back problems but one of the possible phenotypic expressions of an inherent weakness? In other words, are the circumstances that bring about the onset of illness in general as well as back problems more or less the same?

Back problems have been shown to commence early in life [14] and early onset predicts further problems [15-17]. It is therefore important to concentrate the search for risk factors in the younger population so as to eventually be able to develop a primary prevention strategy.

We obtained a data set from the Swedish Army that offered an unprecedented opportunity to conduct a preliminary inquiry into such questions. It consists of cross-sectional data on an almost complete cohort of 18-year old men who had been summoned to serve in the military. About 48,000 individual cases were represented in the data for the year we analyzed (1998), which is an ample enough study population to take into consideration a variety of factors. Selection bias in these data is also minimized because military service is obligatory in Sweden and the full spectrum of society is called to serve. It is also important to note that data were collected prior to the selection process (accept/reject) had taken place, hence eliminating selection bias. Furthermore, almost all in the study population (91%) were still in school at the time they were summoned, which allows analysis of possible factors implicated in back problems prior to exposure to heavy physical workloads entailed in some occupations.

In short, the primary aim in this investigation was to explore how different types of factors are associated with the dependent variables back problems and ill health ("outcome") prior to entry into the labor force. Accordingly, we tested the effect on outcome of five independent variables that comprised two anthropometric factors (height and body mass index), two variables that we defined as psychological (intellectual capacity and coping in relation to stress), and one social variable (type of education). On the basis of these analyses, we then investigated which type of variables was most strongly associated with back problems. In particular, we investigated whether coping in our analysis has so much influence that other factors become negligible. We also compared the pattern of associations for the two outcome variables, back problems and ill health, to indicate whether each is a distinct condition or whether they both might have a common pathogenesis.

In many previous studies, where associations between potential risk factors and back pain were tested and in cases where control for extraneous factors was undertaken, it is difficult to understand how variables entered into the analyses were managed. For example, in most studies relating obesity to low back pain, there is not even a definition of the cut-point for obesity [4]. In our study we decided to follow the recommendations of Zahr [18], to look at the spread of data before deciding on the method of analysis which is most clinically relevant. For example, it would not be suitable to simply divide an independent variable into high and low or to use mean values, if the association is U-shaped or unevenly distributed. The large sample size in our study made it possible to include relevantly sub-classified variables in the analyses.

## Methods

### Study subjects

At age 18, all Swedish male citizens are notified of their military obligation, and most are examined for military suitability the same or following year. In addition, a small number of naturalized Swedish males aged 18–24 are summoned for the enrolment test at the time of their naturalization. Only a small proportion of subjects – in 1998, the year of our study, 2%- are rejected before appearing at the enrolment center. These cases consist of imprisoned subjects and those who obviously cannot join the military force because of serious disability (such cerebral paresis). These are the only subjects not participating in the enrolment procedure. Of the other 48,502 who were summoned, 76% were aged 18, 22% were 19, and the remaining 2% were aged 20–24.

### Enrolment procedure

The enrolment procedure takes place throughout the year in one of five regional recruitment centers. Physical and psychological tests are conducted at these centers to determine both ability to perform military or civil service and type of service for which individuals are best suited. The procedure consists of a series of self-administered questionnaires, physical and medical examinations, and semi-structured psychological interviews. Each aspect of the testing procedure is undertaken independently of the others, and examiners are blind to the results of previously administered tests.

A determination about whether individuals are inducted is based on a scoring system that signifies a prognosis of ability to perform a worthwhile job in the military. All were subjected to an identical screening process, with the exception that, if at some point in the test procedure an individual's unsuitability for military service became apparent, no further tests might be administered, if it was obvious that the person would be rejected from military service. This would typically happen in some subjects who at the medical examination were found to be medically unfit, or in those who at the intellectual capacity test were found to be definitely mentally substandard. Therefore, the number of persons participating in each test varies somewhat throughout the report.

### Variables of interest

Variables for this study were derived from the battery of test procedures taking place at enrolment. Each of these variables was collected independently of the others and without access to previously collected information. Both the independent and the dependent variables are described below:

#### Independent variables (in order of the test procedure)

▪*Type of education *is obtained with a questionnaire completed at the test center. Educational programs in Sweden vary in contents and difficulty. The choice of and acceptance into study programs in the last 3 years of schooling depend on previous school performance. The study programs for the last 3 years of schooling may be grouped into three broad classes: a) the natural sciences, which offers the greatest opportunities to continue directly into further higher studies, b) the social sciences/humanistic studies, which offer somewhat fewer opportunities for further higher studies, and c) "other studies" which include a broad spectrum of courses that not only lead to various trades/skilled jobs but are aimed also at keeping the less intellectually inclined occupied in a meaningful way. c) The "other studies" educational track provides only a few if any opportunities for further higher education. Included in the "other" group are also 4,236 young men not placed in the first two educational classes because they had only 9 years of education or they were refugees/immigrants whose educational status was unclassifiable. Separate analyses for this foreign subgroup revealed that its profile matched well with that of the whole group of "other studies".

▪*Body height *is measured by the medical personnel during the medical examination as well as *body weight *and *body mass index *(*BMI*) is calculated from body weight (kg) and height (cm).

▪*Intellectual capacity *is determined at the time of enrolment with a battery of tests. It consists of a total of 200 items, with separate sections on logical, verbal, spatial, and theoretical/technical aspects of reasoning. Individual test scores are summed into a final score, which in turn is divided into 9 subgroups, with 1 being the lowest and 9 the highest, the so-called stanine scale. Some subjects, already classified as obviously medically unfit for military service, will not reach this test station.

▪Psychological strength (*coping with stress*) is obtained from a structured interview with a psychologist. Based on the young men's recollected responses to past events, this variable is intended to reflect the level of adaptation in life, including psychological and physical endurance under stress. Its ultimate aim is to determine ability to cope during war-like situations. Some subjects, already classified as obviously medically or intellectually unfit for military service, will not reach this station.

#### Dependent (outcome) variables

A health-profile is obtained through a medical interview/examination procedure, based on a health-declaration by the enrollee. The health-declaration questionnaire, filled out at home before travel to a regional enrolment center, consists of information on health problems that are of importance in evaluating future performance as a soldier. Enrollees are also requested to bring copies of their previous medical records and other health-related documents to the enrolment center. At the enrolment center, they go through the questionnaire with a nurse, who also performs a brief physical exam to document obvious disease or impairment. A physician performs another, more thorough, anamnesis and exam. Afterward, with data from the various sources, the physician diagnoses any health problems. Diagnoses are coded in accordance with the WHO International Classification of Diseases, ICD10 categories.

In this study, a number of diagnoses are defined as "*back problems*" (M40–M55 according to the ICD10 classification system), including non-specific back or neck pain/disease. No consideration in our study was made of the "severity" of the diagnosis. Individuals who indicated that they had a back problem were "forced" into one of these diagnoses by the medical practitioner, although the problem in reality very well might have been of non-specific character.

*Ill health *was defined as any type of medical condition that was considered by the medical practitioner to be sufficiently to make military service unwarranted.

### Validity aspects

The study sample represents almost the whole 1998-cohort of 18-year old Swedish men (those 18-year olds who are not tested at the time they are summoned are tested the following year, thereby excluding the possibility of selection bias). The minute fraction (2%) who was not summoned to the test procedure was usually institutionalized for reasons of criminality or severe disability. Whether this resulted in a bias in terms of the associations tested in this study is not known, but the group is too small to be able to influence the results to a large extent. Individuals who did not complete the whole screening procedure are all found in the disadvantaged groups (poor health and/or low intellect), and – had they reached the psychological test station – are likely to also have poor coping skills). Their absence from our analyses would therefore result in an underestimation of the strength of associations rather than the opposite.

When recruits have completed the battery of tests and examinations, data are sent from the five regional test centers to a central data office for cleaning and storage. The quality of data is also checked and if data problems are found, including unusual reporting patterns from the medical and psychological examinations, regional centers are notified. The data-base is well maintained and repeat comparisons are made with previous years to assure standardization. No abnormalities were observed for the data from the year of our study and values for the examined cohort are consistent with the pattern observed over the years.

The Swedish educational system is uniform, and data from it are classified according to an official coding system and are unlikely to be misclassified.

The test instrument used to determine intellectual capacity is performed on a personal computer, computer-analyzed, and scored prior to and independently of any other test administered during the enrolment procedure. The test of intellectual capacity is unavailable to the public. It has been used by the Swedish army for several decades, however, and is under continuous scrutiny and development in order to meet the needs of the military organization. Scores obtained from this instrument have been shown to be relatively normally distributed [19], as is confirmed in our study. According to a validation study carried out on a sample of 15,195 young men of different types and abilities, scores from the present version of the test have been found to be well correlated with military grades at the time of completion of military service [20].

The coping variable is obtained through structured, standardized interviews, unavailable to the public, conducted by psychologists trained for this task. Feed-back from central office on its results is provided to the individual testing centers if any deviations from the national pattern are noted. Because not all individuals reach this stage of the induction process, some deviation from the perfect norm would be expected, as confirmed in our study. Coping determined in this manner also has been validated as explained in the paragraph above and found to correlate well with grades at completion of military service [20].

### Analysis and presentation of data

#### Transformation of independent variables

Intercooled STATA 7.0 was used for the statistical analysis. Each of the 5 independent variables (*height, BMI, type of education, intellectual capacity*, and *coping*) was tested against the outcome variables (*back problem*s and *ill health*) using the polynomial regression method so as to determine the maximum power with statistical significance (up to the 4^th ^power) of the polynomial [18]. We used this procedure to identify whether associations follow a linear, quadratic, cubic or quadric pattern, or whether there is no pattern at all. This information was used to categorize the independent variables in a clinically relevant manner and to reduce data information loss.

#### Interactions

Presence of effect modification may confuse the overall outcome between two variables. For this reason, potential interactions between the variables in relation to outcome were tested using the basic independent variables as described above. This was done by testing two of these at a time and their product in a logistic regression. The pre hoc level of significance was set at 0.01. In this way, any modification between specific subgroups of one variable in relation to specific subgroups of the other variables could be detected. Presence of effect modification would require data to be reported in the relevant strata, whereas absence of effect modification would allow for crude or adjusted overall estimates. This algorithm of analysis followed the recommendations by Gerstman [21].

#### Bivariate and multi variable analyses

Links between each independent variable and the outcome variables were tested, one by one, and reported as odds ratios. In order to reveal any associations between these variables, they were then tested against each other, except height and BMI, which by definition are dependent on each other.

Independent variables that were associated with each other were then introduced into the analyses one at a time, controlling for individual confounding. Thereafter, the association for each independent variable was again tested against the outcome variable, whilst controlling for all the other independent variables. Separate analyses were made for height and BMI, as BMI is a product of weight and height. Finally, the area under the receiver operated curve (ROC) was identified. This area denotes the proportion of individuals classified using a specific model. Values between 0.5 and 1 indicate an increasingly higher ability to differentiate true cases from true non-cases.

Due to the large study sample, confidence intervals were so small that they became meaningless to report and even small associations would be statistically significant making also p-values meaningless. The size of the estimate was therefore taken into account, rather than the size of the p-value.

## Results

### Study subjects

In all, 48,502 young men went through all or part of the enrolment process. All of these went through the medical examination, 46,097 were also submitted to the test for intellectual capacity, and 40,094 of the original group reached the psychological interview.

The most frequently diagnosed problem was related to the musculoskeletal system and connective tissues (21%), followed by problems with the respiratory organs (19%), and psychosocial problems (17%). In the whole cohort, 5450 individuals (11%) were noted to have a current back problem. These consisted of individuals whose back problem was thought to interfere with military service, in other words they would represent cases of more serious back problems than mere "any back problem in the past or present", commonly used in other epidemiologic studies. Table 1 lists the main specific diagnoses that compose the category of "back problem" (BP) and the number of young men with each specific diagnosis.

In all, 28% were classified as having "ill health" (IH), i.e. to be unsuitable for military service. The reason for this could be single or multiple diagnoses. Among those with a back-related diagnosis, at least 6% were in this category. However, because only one reason for IH usually was recorded statistically, it was not possible to specify how many of the back problems were serious enough to exclude military service. Of those with BP, 42% were considered unsuitable for military service, i.e. mostly for reasons other than the back. The corresponding figure was 31% for the 42,053 individuals without a reported BP.

Twenty-three percent of the participants received a natural science education, 18% attended humanistic/social studies, and 59% were classified under "other studies". The independent variables height, BMI, intellectual capacity and coping are described in Table 2 for the initial sample.

### Grouping of independent variables

The patterns of associations between the five independent variables and the two outcome variables resembled each other. Most of the independent-outcome variable patterns were 2^nd ^degree polynomous (for example U-shaped) showing that a dichotomous separation or the reporting of mean values would result in loss of data information. Therefore, height was transformed into quartiles, BMI into the standard subdivision of underweight (<18.499), normal weight (18.5–24.999), overweight (25.0–29.999), and obesity (≥ 30.0). For intellectual capacity and coping suitable subdivisions were observed at the scores of 1–3 ("low"), 4–6 ("medium"), and 7–9 ("high"). The 3 main educational groups were maintained.

### Testing for interactions

No significant interactions between any of the independent variables could be identified.

### Associations between the independent variables

There were, however, definite associations between all five independent variables. In summary:

• Height was positively associated with type of education, intellectual capacity and coping.

• Normal BMI was associated with natural science studies, high intellectual capacity and high coping scores. Being underweight was also associated with high intellectual capacity scores and a natural science education but was not associated with high coping scores. Heavy overweight was associated with "other" education, low intellectual capacity scores and low coping scores.

• Coping and intellectual capacity were strongly positively associated with each other.

• There were dose-response associations between coping and type of education for the low and high coping subgroups.

• There was a very strong dose-response association between intellectual capacity and type of education, a result that was predetermined because intellectual capacity inheres in the process of selection for the different types of education.

Because all independent variables were associated with the outcome variables and with each other, confounding was a possibility.

### Associations between the five independent variables and back problem

The unadjusted odds ratios for the 5 independent variables in relation to BP are described in column 1 in Table 3, and a further description is provided below.

BP was somewhat more common among the tall. The underweight were more likely to be classified as having BP than those with normal- or overweight. There were negative associations between back pain and education, intellectual capacity, and coping, with odds ratios ranging from 1.4 to 6.5.

The all-variables adjusted odds ratios are found in the second column in Table 3. Coping remained strongly negatively associated with BP (odds ratio approximately 7) with none of the others exceeding 1.5 (height). In fact the odds ratios of BMI, education and intellectual capacity were all reduced in this model.

A post hoc analysis revealed that the odds ratio for education was reduced from 1.6 to 1.3 when adjusting for intellectual capacity. Interestingly, the initial odds ratios for education (1.6) and intellectual capacity (2.1) were reduced to 1.2 and 1.3, respectively, after adjusting for coping. In other words, intellectual capacity "overruled" type of education but coping had this effect on both education and intellectual capacity. Even so, the area under ROC was only 0.66 (0.65–0.67), for the final model.

### Associations between the five independent variables and ill health

The unadjusted odds ratios for ill health, as described in column 1 in Table 4, reveal that the tallest had a somewhat higher prevalence of IH as well as the underweight and the obese. There were for IH, as for BP, negative dose-response patterns for education, intellectual capacity, and coping. A remarkable odds ratio of 134 was noted for the group with the lowest coping scores.

The all-variables adjusted variables are listed in column 2 in Table 4. Yet again, this shows that it is coping that contributes most to the link with IH with the other four variables not adding much. Only coping remained strongly associated with ill health (odds ratio 104.3). The area under ROC was considerably higher, 0.89 (0.89–0.90), for the final model.

## Discussion

Some of our results were similar to those found in numerous other epidemiologic studies, namely some weak to moderately strong associations between a number of independent variables and BP. Our study, however, is exceptional in certain respects and has produced findings not commonly reported in the epidemiologic literature.

First, we noted that coping is strongly associated with BP and that this is an association that withstands the effect of the other variables, whereas the other variables that we tested typically became weakened when challenged with coping. We thus surmise that coping may not be a mere risk indicator, whereas the others most certainly are.

Second, we found that the statistical pattern is very similar for BP and IH in general. This finding suggests common aspects in the pathogenesis of both. In fact, it may imply that BP is but one of the expressions of IH in general. Coping, with an exceptionally high odds ratio, covers a large area under ROC for IH but only a small area in relation to BP. This, again, indicates that young men with BP may constitute a subpopulation nested within the group with IH.

These findings raise the question of *why *inability to cope is associated with BP and IH IH. We suggest three possibilities, with one not necessarily precluding the other. Regarding the first possibility, poor coping skills may serve to impede social and educational achievement. Early tracking and negative experiences of this nature would be a basis for a pervasive sense of powerlessness in social relations and a sense of not being in control of one's destiny–in short, self-recognition of being in the lowest social stratum, reliant upon the will of others. Studies from the literature on general health and illness have shown that a sense of powerlessness is associated with wear-and-tear on the body, diverse types of ill health, and self-reported poor health (22,23). The other possibility is that ill health may have a deleterious effect on coping, which becomes particularly apparent when examining young men's beliefs on their own ability to cope in war-like situations. The third possibility is that inability to cope and ill health may have a common origin, such as a genetic predisposition toward general frailty. Within the wider population, a certain subgroup may, consequently, be more prone toward diseases of various types, including back problems.

Methodologically, our study is unique in that it consists of a large and almost entire cohort, which excludes selection bias and allows for meaningful multi variable analyses. In addition, our cohort consists of young men, and data from them were collected prior to their entry into the work force, which enabled us to avoid the confusing element of occupational exposure. Finally, as stated earlier, the various variables in the study were collected independently of each other, and, it is important to note, the psychologists who assessed the inability to cope were blinded to data on health, including back problems. For this reason, their findings would have remained uncontaminated by the pre-existence of BP and IH.

Bias might be suspected in a study in the military sector. Conceivably, some of those called to serve may resist induction, and, in doing so, they may over-report disease and otherwise attempt to subvert induction tests. For the most part, however, we found dose-response relationships between the outcome and independent variables, which would be unlikely, had willfully poor performances subverted test scores to a substantial extent. Also, the supply of young men at enrolment is far larger than the demand for manpower, with only less than half of those who went through the enrolment procedures in 1998 actually later being inducted into the military. For this reason, over-reporting of ill health in order to avoid military or civil service is hardly necessary. Willingness to serve in the military varies over time, but military forces considered it to be quite high when data for this study were collected. Under-reporting of ill health by some young men intent on a military career may therefore be more likely.

This study, however, has two notable weaknesses. First and obviously, our cross-sectional data collected at one point in time cannot be used to test a cause-effect but mere associations (24). Second, although the most important independent variable, coping in relation to stress, has been found to be a valid predictor of soldiers' performance during their military service (20) its exact contents and definition are unknown to us. If the Swedish Army defines coping in relation to physical stamina in war-like situations, then the presence of certain diseases, such as asthma, might induce a non-coping response. In other words, ill health and poor coping may be but expressions of the same phenomenon. This might explain the very high association between coping and IH.

Interesting as our results may be, they cannot really be taken seriously, until the coping variable is properly defined and described but, unfortunately, the Swedish Army is not able to divulge its interview questions. Obviously, the link between coping and BP/IH must be tested with a known coping instrument. It is also always necessary to challenge the results of any study in other settings. We therefore proceeded to conducting a prospective observational study of the development of back problems in military recruits, who were investigated for coping ability prior to the first three months of military service, using a well known coping questionnaire. The results of this second study, which has been completed, will be reported separately.

## Conclusion

Our conclusions, at this time, are:

• Coping, as defined by the Swedish Army, is strongly associated with back problems in young Swedish men.

• In this cohort, coping, as defined by the Swedish Army, is very strongly associated with ill health.

• In this cohort, coping, as defined by the Swedish Army, has a strong effect on the association between height, body mass index, intellectual capacity, type of education and the outcome variables back pain and ill health.

• In this cohort, back pain and ill health, as defined by the Swedish Army, have a similar pattern of associations.

• The "black box" of coping, as defined by the Swedish Army, must be defined and described.

• These findings need to be reproduced in prospective studies, in order to test for causality.

## Competing interests

The author(s) declare that they have no competing interests.

## Authors' contributions

Ingvar Ahlstrand participated in the data collection, was the resource person in relation to the screening process, supervised the military data base, was responsible for its quality control and provided the data. He read and commented the various drafts of the manuscript.

Charlotte Leboeuf-Yde formulated the research questions, performed the preliminary analyses, laid the strategy for the secondary analyses, participated in the secondary data analyses, wrote the methods and results sections and was co-author on the rest of the manuscript and was the main responsible for the research group.

Kristian Larsen performed the secondary analyses, participated in the data interpretation and read and commented each draft.

Ernest Volinn participated in the strategy for the data analysis, assisted in the data interpretation, wrote the first drafts of the introduction and discussion sections, read and commented on each draft.

## Pre-publication history

The pre-publication history for this paper can be accessed here:



## Figures and Tables

**Table 1:** Description of the most commonly reported back-related diagnoses among those who reported some type of back problem (n = 5450) in a cohort of young Swedish men. More than one diagnosis per person was possible.

**Diagnosis**	**Number of individuals**
Unspecified back pain	3553
Unspecified back disease	1171
Unspecified scoliosis	251
Lumbago (excluding sciatica and discal hernia)	156
Cervicalgia (excluding neck pain from discal disease)	98
Unspecified lordosis	44
Juvenile idiopathic scoliosis	41
Juvenile spinal osteochondrosis (Calve's or Scheuermann, excluding postural kyphosis)	28

**Table 2:** Description of the independent variables in a cohort of young Swedish men. Height and BMI are continuous variables, whereas intellectual capacity and coping are interval variables but treated as continuous variables in this analysis.

**Variable**	**Numbers tested**	**Range of Values**	**Mean; SD**	**Skewness**	**Kurtosis**
Height	48,502	128–208	179.7;6.7	0.0	3.2
BMI	48,502	14.3–54.6	22.5;3.4	1.5 (0.9)*	7.4 (4.5)*
Intellectual Capacity	46,097	1–9	5.0;2.0	0.0	2.4
Coping	40,094	1–9	4.8;1.8	-0.1	2.2

* after log transformation

**Table 3:** Unadjusted and adjusted odds ratios for associations between each independent variable and back problem in a cohort of young Swedish men. The number of study subjects in the bivariate analyses are shown in the first column and the number of participants in the multivariate analyses was 40,094.

**Independent variable (N)**	**Unadjusted odds ratio**	Odds ratio adjusted for all variables**
**Height**		
1(shortest) (12,022)	1 (index)	1 (index)
2 (12,835)	1.0	1.1
3 (9,820)	1.1	1.3
4 (tallest) (10,592)	1.2	1.5
		

**BMI**		
underweight (2,877)	1.5	1.2
normal weight (34,104)	1 (index)	1 (index)
overweight (6,487)	1.0	1.0
obese (1,801)	1.0	0.8

**Intellectual capacity**		
high (10,623)	1 (index)	1 (index)
medium (24,766)	1.5	1.2
low (10,809)	2.1	1.2

Education*		
high (11,108)	1 (index)	1 (index)
medium (8,582)	1.4	1.2
low (28,719)	1.6	1.2
		

**Coping**		
high (7,835)	1 (index)	1 (index)
medium (21,800)	3.3	3.2
low (10,459)	6.5	6.9

*Education is divided into high (natural sciences/mathematics), medium (human sciences/language) and low (vocational studies/any other) based on the opportunities these educational strands provide for further studies.

** Height and BMI are not adjusted for each other as they are interdependent.

**Table 4:** Unadjusted and adjusted odds ratios for associations between each independent variable and ill health in a cohort of young Swedish men. The number of study subjects in the bivariate analyses are shown in the first column and the number of participants in the multivariate analyses was 40,094.

**Independent variable**	**Unadjusted odds ratio**	Odds ratio adjusted for all variables **
**Height**		
1(shortest) (12,022)	1 (index)	1 (index)
2 (12,835)	1.0	1.1
3 (9,820)	1.1	1.1
4 (tallest) (10,592)	1.4	1.0
		

**BMI**		
underweight (2,877)	1.6	0.9
normal weight (34,104)	1 (index)	1 (index)
overweight (6,487)	1.1	0.9
obese (1,801)	2.1	0.9

**Intellectual capacity**		
high (10,623)	1 (index)	1 (index)
medium (24,766)	1.4	1.0
low (10,809)	4.0	1.4

Education*		
high (11,108)	1 (index)	1 (index)
medium (8,582)	1.4	1.0
low (28,719)	2.7	0.9
		

**Coping**		
high (7,835)	1 (index)	1 (index)
medium (21,800)	2.8	2.7
low (10,459)	134.3	104.3

*Education is divided into high (natural sciences/mathematics), medium (human sciences/language) and low (vocational studies/any other) based on the opportunities these educational strands provide for further studies.

** Height and BMI are not adjusted for each other as they are interdependent.
